# Effects of *Lactobacillus plantarum* on intestinal integrity and immune responses of egg-laying chickens infected with *Clostridium perfringens* under the free-range or the specific pathogen free environment

**DOI:** 10.1186/s12917-020-2264-3

**Published:** 2020-02-07

**Authors:** Tianyue Xu, Yan Chen, Longfei Yu, Jun Wang, Mingxing Huang, Nianhua Zhu

**Affiliations:** Present address: Jiangxi Province Key Laboratory of Animal Nutrition, College of animal science and technology, Jiangxi Agricultural University, Nanchang, Jiangxi 330045 People’s Republic of China

**Keywords:** Chickens, *Lactobacillus plantarum*, *Clostridium perfringens*, Environment, Intestinal injury score, Inflammatory cytokines

## Abstract

**Background:**

Necrotic enteritis, which is caused by *Clostridium perfringens*, has resulted in more than $2 billion losses in the poultry industry every year. Due to the ban of antibiotics in feed industry, alternatives like environment improvement and probiotics have been found to be effective as well. In our study, we aim to explore the protective effect of *Lactobacillus plantarum* supplementation on *CP* infected chickens in two environments.

**Results:**

The results showed that the *Clostridium perfringens* administration led to visible and histomorphological gut lesions. In the specific pathogen free or free-range system environment, dietary supplementation with *LP* obvious increased the ratio of intestinal villus height to crypt depth and the expression of *MUC2* mRNA in ileum mucosa, then reduced the mRNA expression level of *TNF-α* gene in the ileum mucosa. *LP* treatment significantly reduced the contents of total protein, total superoxide dismutase and glutamic oxaloacetic transaminase in serum of the chickens.

**Conclusions:**

The specific pathogen free environment contributed to the recovery of pre-inflammation of the chickens, and free-range system environment contributed to the repair of damage in the later stages of chicken inflammation. Supplementation of *LP* in FRS environment was more conducive to the recovery of *CP* infected in chickens.

## Background

Necrotic enteritis (NE), which is caused by *Clostridium perfringens* (*CP*), is one of the most important enteric diseases in the global poultry industry which has resulted in about 6–7 billion US dollars loss per year [26]. *C. perfringens* type A is a gram-positive, spore-forming, rod-shaped bacterium, which is the main pathogen that causes clinical and subclinical necrotic enteritis in poultry [39, 42]. The key factor for the development of NE is the change of gastrointestinal environment, which creates favorable conditions for the growth of *CP*. Environment improvement (intensive feeding patterns) and the extensive use of antibiotics have played an important role in preventing *CP* infection and the incidence of NE in the past decades [33]. Antibiotics are considered to be effective measures to reduce the incidence of NE, but due to ban on feeding growth-promoting antibiotics in Europe, there has been an increase in the incidences of NE [14]. Owing to the ban on the use of antibiotics in feed industry, researchers have searching for alternatives to help growth-promoting and prevention of the incidence of NE.

The European Union (EU) has completely banned the use of traditional laying hens from January, 2012 [35]. Laying hens are raised in large cages, with self-owned free-range feeding, shed flat feeding and organic feeding and other better ways of poultry breeding. Improving animal welfare is the key to improving bird health, quality of life and productivity [2]. A diet of mealworms and fresh grass contribute to improve gait score, chicken meat quality, produce higher platelet values and richer intestinal microorganisms in free-range environment [22]. Free-range environment can improve egg laying performance, promote feeding activities of chickens, and improve animal welfare. Specific pathogen free (SPF) animals were introduced in the 1960s to reduce disease or infection as a variable not required in their experiments [28]. Today, the overall strategy for most livestock industries is to incubate animals that do not contain infectious agents from sterile livestock or cesarean delivery aseptic techniques for experimental animal models of various diseases [11].

Probiotics are defined as a class of active microbial supplements that are beneficial to the host, improving host intestinal microbial balance and beneficially affecting the host [9]. A large number of studies have shown that probiotics have a variety of biological functions. They can produce molecules with antimicrobial activity, target specific pathogens, and even inhibit the adhesion of pathogens. They can also improve intestinal morphology, maintain intestinal microbial balance, and interact with host to improve immunity [9, 10, 18]. *Lactobacillus plantarum* and *Lactobacillus casei* are the most commonly used probiotics. Studies have shown that *Lactobacillus casei* can reduce the incidence of diarrhea, and interact with human mucosa, significantly reducing the release of inflammatory factors in Crohn’s disease [8, 13, 29]. Studies have shown that *LP* can improve the growth of weaned piglets, promote the development of small intestine villi, and increase serum IgM, IL-10 and TGF-β levels [30].

The purpose of our experiment is to study the effect of *LP* supplementation on immune function of chickens after infected with *CP* in different environments. We aim to explore whether different feeding methods and probiotics can alleviate the adverse effects of necrotizing enteritis by *CP* on chickens. We also aim to provide effective measures and a theoretical basis to aid in reduction of losses from NE in the poultry industry.

## Results

### Intestinal lesion score and immune organ index

**Fig. 1:**
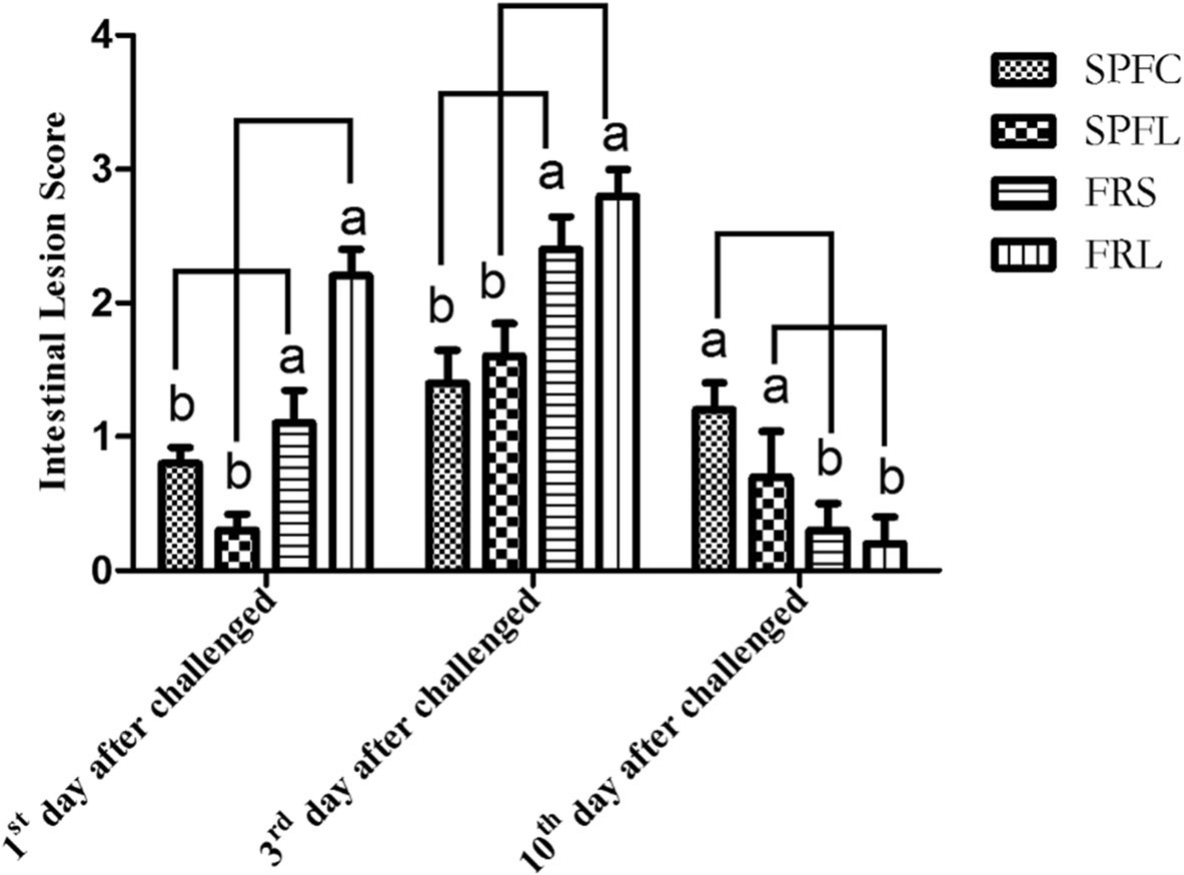
The effect of *Lactobacillus plantarum* and environment on the ileum injury score The different letters (a, b) of linear connection showed significant difference (*P* < 0.05)

There was no death of chickens in the whole experiment. Compared to the SPF environment, chickens fed in FRS environment had a higher intestinal lesion score at 1st and 3rd day after exposure to *CP* (*P* < 0.01, Fig. 1), and then had lower score at 10th after *CP* administration (*P* < 0.05). *LP* had no significant effect on ileum injury score. All chickens administered with *CP* had different degrees of ileum injury. FRS environment inflammatory response was more harmful in early stages, and recovery was faster in the later stage of inflammation, which indicated that the FRS environment was more conducive to the recovery of chickens.

The immune organ index of chickens after administration with *CP* are shown in Table 3. SPF environment significantly increases the thymus index in the 1st and 10th day after *CP* administration (*P* < 0.05). FRS environment increased the bursa index in 1st day after chickens were administered with *CP* (*P* < 0.05). *LP* treatment had an increasing trend on thymus index in 10th day after *CP* exposure. The results indicated that the SPF environment enhances immune function by increasing the chicken thymus index, while the FRS environment enhances immune function by increasing the chicken bursa index.

### Observation and analyses on intestinal morphology

Difference of feeding environment and *LP* treatment on duodenal morphology of chickens after *CP* administration is showed in Table 4. Compared to SPF environment, the FRS environment can significantly increase the duodenal villus height (*P* = 0.035, on 3rd day after *CP* administration), the depth of crypt (*P* < 0.01, on 1st, 3rd day after *CP* administration), the V/C (*P* = 0.005, on 10th day after *CP* administration). *LP* treatment increased the villus height and V/C (*P* < 0.05). Under the experimental conditions, the environment and *LP* had an interaction effect on the duodenal villus height (*P* = 0.038, on 10th day after *CP* administration), the depth of the crypt of the duodenum (*P* < 0.05), and the V/C (*P* < 0.01, on 1st and 10th day after *CP* administration).

Difference of feeding environment and *LP* treatment on jejunal morphology of chickens after *CP* administration is shown in Table 5. Compared to FRS environment, the SPF environment can significantly increase the jejunum villus height (*P* < 0.01, on 3rd and 10th days after *CP* administration), and the V/C (*P* < 0.01). *LP* treatment had an increasing effect on the V/C (*P* < 0.01). Under the experimental conditions, the environment and *LP* had an interaction effect on the jejunum villus height (*P* < 0.05), the depth of the crypt (*P* < 0.05, on 1st day after *CP* administration), and the V/C (*P* < 0.01, on 1st and 3rd days after CP administration).

Difference of feeding environment and *LP* treatment on ileal morphology of chickens after *CP* administration is shown in Table 6. Compared to SPF environment, the FRS environment can significantly increase the ileal villus height (*P* < 0.01), and the depth of the crypt (*P* < 0.01). *LP* treatment increased the V/C (*P* < 0.01, on 1st and 10th days after *CP* administration). Under the experimental conditions, the environment and *LP* had an interaction effect on the ileal villus height (*P* < 0.01, on 1st and 3rd days after *CP* administration), the depth of the crypt (*P* < 0.05, on 3rd and 10th day after *CP* administration), and the V/C (*P* < 0.01, on 1st and 3rd days after *CP* challenged).

### Serum biochemical and immune parameters

The results of serum biochemical indicators of chickens are shown in Table 7. Compared with the FRS environment, the SPF environment can significantly reduce the TP levels in the serum of chickens (*P* = 0.038, on 10th day after *CP* administration) and AKP levels (*P* < 0.05, 3rd and 10th days after *CP* administration). However, on 1st day after *CP* administration, the FRS environment can significantly reduce the levels of GOT in the serum of chickens (*P* = 0.001), and the T-SOD has a tendency to decrease (*P* = 0.096). *LP* treatment group can significantly reduce the levels of TP, T-SOD and GOT in the serum of chickens (*P* < 0.05). Under the conditions of this test, the environment and *LP* had an interaction effect on TP (*P* = 0.002, on 3rd day after *CP* administration) and AKP (*P* = 0.045, on 10th day after *CP* challenged).

The results of chicken serum immune index are listed in Table 8. Compared with the FRS environment, the SPF environment can significantly increase the serum IgA (*P* = 0.044, on 10th day after *CP* administration) and IgM (*P* = 0.041, on 1st day after *CP* administration) levels in chickens. The *LP* treatment group can significantly increase the serum levels of IgA (*P* < 0.05, on 3rd and 10th days after *CP* administration), IgM (*P* = 0.010, on 10th day after *CP* administration) and IgG (*P* < 0.05, on 3rd and 10th days after *CP* administration).

### Gene expression of ileal mucosal cytokines and tight junction proteins

The expression results of the tight junction protein gene in the ileal mucosa of chickens are shown in Table 9. The *LP* treatment group can significantly promote the mRNA expression of *MUC2* gene (*P* < 0.05, on 1st and 3rd days after *CP* administration) and *Claudin* gene (*P* = 0.041, on 3rd day after *CP* administration), and promoted mRNA expression of *MUC2* gene (*P* = 0.072, on 10th day after *CP* administration) and *Occludin* gene (*P* = 0.082, on 3rd day after *CP* administration). There was no significant difference on tight junction proteins between SPF and FRS environment. Under the conditions of this test, the environment and *LP* had trends in interaction effect on the mRNA expression of *MUC2* gene (*P* = 0.060, on 1st day after *CP* administration).

The results of gene expression of cytokines in ileal mucosa of chickens by *LP* and environment are shown in Table 10. Compared with the FRS environment, the SPF environment has a tendency to decrease the expression of *IL-1β* gene mRNA (*P* = 0.086, on 3rd day after *CP* administration). *LP* treatment group can significantly reduce the expression of *TNF-α* gene (*P* = 0.022, on 3rd day after *CP* administration) and *TLR4* gene (*P* = 0.039, on 10th day after *CP* administration) mRNA in the ileal mucosa of chickens.

## Discussion

*CP* is the main pathogen of avian NE and can also cause human gas gangrene and food poisoning [5]. As mentioned earlier, a loss of more than $2 billion in poultry industry each year, is attributed to necrotizing enteritis which is caused by *CP*. In order to reduce losses, many measures (improve of the feeding style or environment, additive probiotic, i.e.) are used to competitively inhibit the growth of *CP*, improve the defense function of intestinal viscosity, and increase the immune function of chickens. Thymus is the main lymphoid organ in poultry, which is responsible for producing a variety of immune T cells [32]. Bursa of Fabricius is the main site of immunoglobulin synthesis, which plays an important role in cell-mediated immunity. The increase of thymus weight and relative weight of bursa of Fabricius (immune organ index) can reflect the increase of lymphocyte level. Kikuchi Y (2014) research shows that *Lactobacillus plantarum* can promote the proliferation and differentiation of B and T cells and improve the immune function of mice [21]. Our results show that SPF environment could increase the thymus index, and reduce the intestinal lesion in early stage of *CP* infection, while the FRS environment had beneficial effect on recovery in the later stage, and increased the bursa index in early stage.

Studies have shown that chickens infected with *CP* had a higher ileal injury score than unaffected chickens [6]. In our study, *LP* had no significantly effect on ileum injury score, which may be due to the feeding time of *LP* supplementation. However, SPF environment was conducive to early recovery of inflammatory response. FRS environment inflammatory response was more harmful in the early stage, and recovery was faster in the later stage of inflammation which indicated that the FRS environment was more conducive to the recovery of disease in chickens. This may be due to the fact that chickens in SPF environment were raised in isolators, and the air in SPF environment was cleaner than that in FRS environment. FRS environment had a great influence on the later stage of inflammation in chickens, which was because FRS environment can reduce fear, promote foraging activities of chickens, improve animal welfare, and enhance immune function through positive emotional state [2].

Gut microbiota can affect intestinal morphology through modifications of villus height and crypt depth [4]. *CP* can directly damage intestinal mucosa, alter intestinal microflora, or damage the immune system [41]. The most obvious macroscopic lesions can be seen in the small intestine, the duodenum, the jejunum and the ileum, and sometimes the cecum, which are thin-walled, fragile, dilated and filled with gas [38]. Our results show that SPF environment and *LP* can significantly increase the ratio of villus height and crypt depth in duodenum and jejunum of chickens, while FRS environment can significantly increase ileal villus height and crypt depth in chickens. Increasing crypt depth might contribute to intestinal regeneration and recovery of NE infected chickens. This is consistent with previous studies [20, 30]. Studies have also shown that the intestinal microflora of chickens in FRS environment is more complex [1]. *Lactobacillus plantarum* had antagonistic activity against pathogenic microorganisms and spoilage microorganisms by producing organic acids, bacteriocins, and bacteriocin analogs [19, 27, 36, 37]. *Lactobacillus plantarum* can promote the development of small intestine villi, and supplementation with *Lactobacillus acidophilus* in the diet had a tendency to increase the height of the small intestine villi [25]. The results obtained herein mean that *LP* has the ability to maintain mature and functionally active epithelial cells. These results also suggest that SPF and FRS environments may effectively mitigate NE-induced intestinal injury by improving intestinal integrity, intestinal morphology and intestinal microbial balance in chickens.

After chickens were infected with *CP* or on the subclinical infection of NE, the small intestine was damaged [12, 23]. The intestinal injury may cause *CP* to reach the bile duct and portal vein blood flow [24]. The association with hepatitis or biliary hepatitis [17] resulted in rise of the levels of T-SOD, AKP and GOT in serum of chicken. Our experiment showed that *LP* treatment reduced the content of T-SOD, GOT and AKP, and increased the levels of IgA, IgM and IgG in the serum of chickens. These results mean that *LP* could restore the antioxidant capacity and improve immune function of chickens. In addition, IgA in poultry was present in most intestinal cells. The release of sIgA into the intestinal cavity through transepithelial transport and the neutralization or prevention of pathogen binding to the mucosal surface were widely considered to be essential to protect the mucosal surface from toxins, viruses and bacteria [40].

Some bacterial pathogens can impair intestinal barrier function by disrupting tight junctions [31], such as *CP*, *Clostridium difficile* [34]. *IL-1β* was a major pro-inflammatory cytokine that induced its own expression and expression of other pro-inflammatory cytokines, such as the chemokine *TNF-α*, which triggers inflammatory response by activating TLR-mediated signaling pathways [16]. Previous studies have shown that the mRNA expression of *IL-1β* will increase in the jejunum of chickens infected with *CP* [25]. Meantime, our experimental results showed that *LP* treatment increased the mRNA expression of *MUC2* gene, *Claudin* and *Occludin* gene in ileum mucosa of chicken. The *LP* treatment group can also significantly reduce the expression of *TNF-α* gene (*P* = 0.022, on 3rd day after *CP* administration) and *TLR4* gene (*P* = 0.039, on 10th day after *CP* administration) mRNA in the ileum mucosa of chickens. These results suggested that *LP* can protect intestinal epithelial barrier integrity from intestinal pathogens adhesion and invasion and improve intestinal health of *CP* challenged chickens. This indicates that the SPF environment was conducive to the recovery of inflammatory response induced by *CP*, showing that the *LP* treatment group could inhibit the colonization of *CP* in the ileum and restore the intestinal inflammation caused by *CP*.

## Conclusion

In summary, the results of our study indicate that NE impairs the intestinal epithelial barrier of the chickens and induces intestinal injury in *CP* induced model. The supplementation of *LP* in a FRS environment is more conducive to enhancing the adhesion of *LP* in the chicken small intestine, inhibiting the proliferation of *CP*, and thus more effectively controlling or preventing NE. For the early prevention of NE, the risk of chicken disease can be reduced by keeping the feeding environment clean and dry. Our study show that the free-range environment and the supplement with probiotics (such as *Lactobacillus plantarum*) are effective measures to prevent from NE in poultry industry.

## Methods

### Experimental animals, diets, and treatments

One hundred specific pathogen free (SPF) White Leghorns eggs were purchased from Beijing (Beijing Merial Vital Laboratory Animal Technology Co., Ltd.) and hatched in the laboratory. Eighty 1-day-old SPF chickens were randomly divided into four groups of 20 chickens each, a 2 × 2 factorial arrangement of treatments was used in this study:

- Group A (SPFC): SPF environment, animals were fed the basal diet.
- Group B (SPFL): SPF environment, animals were fed the basal diet + *LP* cultures of 10^8^ cfu/kg feed.
- Group C (FRC): FRS environment, animals were fed the basal diet.
- Group D (FRL): FRS environment, animals fed the base diet + LP cultures of 10^8^ cfu/kg feed.

In all the groups, each chicken were orally administered 1 mL *CP* (1 × 10^8^ cfu/mL·day) at 36–42 days of ages (*CP* challenged). After the attacked with *CP*, the chickens were in low spirits, loss of appetite and diarrhea. Corn-soybean meal diets were formulated according to the nutrient requirements for white Leghorn as recommended by National Research Council (1994). The diet composition and nutrient levels are shown in Table 1. All diets were crumbled and powdered. During the whole experimental period, chickens were free to eat and drink water.

**Table 1:** Composition and nutrient levels of basal diet

Ingredients	Content %	Calculated nutrient levels	
Corn	59.04	Metabolizable Energy (MJ/kg)	12.71
Soybean meal	35.04	Crude Protein (%)	21.45
Soybean oil	3.00	Ca (%)	0.87
DL- Methionine	0.10	Available phosphorous (%)	0.36
Choline chloride(50%)	0.05	Methionine + Cystine (%)	0.77
Dicalcium phosphate	1.30	Lysine (%)	1.18
Limestone	1.00		
NaCl	0.30		
Vitamin premix^a^	0.04		
Trace mineral premin^b^	0.10		
mildew preventive	0.03		

^a^ The vitamin premix supplied the following per kilogram of complete feed: vitamin A,12,500 IU; vitamin D_3_, 2500 IU; vitamin K_3_, 2.65 mg; vitamin B_1_, 2 mg; vitamin B_2_, 6 mg; vitamin B_12_, 0.025 mg; vitamin E, 30 IU; biotin, 0.0325 mg,folic acid,1.25 mg; pantothenic acid, 12 mg; niacin, 50 mg

^b^ The trace mineral premin provided the following (per kilogram of diet): manganese, 100 mg; zinc, 75 mg; iron, 80 mg; copper,8 mg; selenium, 0.25 mg; iodine, 0.35 mg

### Preparation of *LP* and *CP*

First, the laboratory *Lactobacillus* (*Lactobacillus plantarum* R1.0320, *LP*) was activated on MRS agar (Aobox, China) plate. *LP* was then mixed and incubated in MRS liquid medium for 24 h alone and the *LP* liquid solution was diluted until the bacterial concentration reached 1 × 10^9^ cfu/mL for feeding test. Type A *CP* was activated on trytose sulfite-cyloserine (TSC) agar plate (Qingdao Hope Bio-Technology Co., Ltd., China), and the anaerobic culture was incubated at 42 °C for 18 h in liquid thioglycolate medium. The cultured bacteria was then diluted until the bacterial concentration reached 1 × 10^8^ cfu/mL.

### Samples collection

After *CP* administration, five birds per treatment (*n* = 5) were randomly chosen from different replicates. For the sample size of each group, we refer to the method of Han et al. (2016) and Bertran et al. (2018) [3, 15], who reported that five chickens in each group were selected for sampling at each time point and then carries out subsequent experimental operations. Chickens were rendered unconscious by intravenously injection of pentobarbital sodium (100 mg/kg body weight) just before slaughter. The chicken jugular vein was cut off and the bleeding blood was collected by collecting blood tubes. The collecting blood tubes were tilted at 45 °and left at rest for 2 h of 4 °C. After the serum was precipitated, it was centrifuged for 10 min at 3000 rpm. The serum was divided into 1.5 ml centrifuged tubes for the detection of serum indexes. Serum biochemical indicators were detected with commercial reagent kits. Serum immune parameters were detected by ELISA method. Dissection was then carried out obtain spleen, thymus, and bursa of fabricius. They were weighed (g) and the immune organ index was calculated. Immune Organ Index = Immune Organ Weight (g) / Live Chicken Weight (g). The duodenum, jejunum and ileum were taken, and rinsed with normal saline, and 2–3 cm of the middle part of the duodenum, jejunum and ileum were then taken, and placed in the 4% paraformaldehyde solution for fixation, and subsequent used for observation of intestinal morphology. The mucosa was scraped from the remaining ileum for inflammatory factor test.

### Intestinal lesion score

Intestinal lesions were scored blindly according to the method of Truscott and Al Sheikhly (1977) [7] with slight modifications. Lesions were scored using a scale from 0 to 3, in which 0 = apparently normal, no obvious damage; 0.5 = severe congestion of serosa and mesentery of small intestine; 1 = thin walled and friable intestines with small red petechiae; 2 = gas in intestinal cavity, needle-like necrosis or ulcer spot in intestinal wall; 3 = gas filled intestinal cavity, patches of necrosis or ulcer in intestinal wall (1 to 2 cm long).

### Intestinal morphological analyses and observation

The duodenum, jejunum and ileum segments that were fixed in 4% paraformaldehyde, were embedded in paraffin. Tissue rings were cut to a thickness of 5 μm and stained with hematoxylin and eosin. The slides were photographed with a Nikon microscope (NIKON INSTRUMENTS (SHANGHAI) CO., LTD. BA210). Villus height and crypts depth were measured from five villi and crypts per slide with the Motic Images Advanced (3.2) software and an average was taken. Villus height was defined as the distance from the villus tip to the villus-crypt junction, and the crypt depth was measured from the villus-crypt junction to the base of the crypt. The mean of villus height and crypt depth were calculated to obtain the villus height-to-crypt depth ratio (VCR). Morphological analyses and observation were conducted at magnifications of 100× for each slide.

### Determination of serum biochemical indicators and immune indicators

Serum indicators were determined according to the manufacturer’s instructions (Nanjing Jiancheng Bioengineering Institute, China). Biochemical indicators are: alkaline phosphatase (AKP), total protein (TP), albumin (ALB), total superoxide dismutase (T-SOD), glutamic oxaloacetic transaminase (GOT). The immune indicators are: IgA, IgM, IgG.

### RNA extraction and cDNA synthesis for gene expression analyses

One hundred milligram of each ileum mucosa was ground in a 1.5 mL centrifugal tube along with 1 mL trizol (Takara Bio, Japan) with an electric homogenizer. The tissues were then fully shaken and placed at room temperature for 5 min. Then, centrifugation was carried out at 12,000 rpm for 10 min at 4 °C. The supernatant was transferred to a new 1.5 mL centrifuge tube, and 0.2 mL of chloroform was added. It was violently shaken for 15 s, and allowed to stand at room temperature for 5 min, followed by centrifugation at 12,000 rpm for 10 min at 4 °C. The supernatant was carefully pipetted into a new 1.5 mL centrifuge tube, and 0.5 mL of isopropanol was added. After mixing well, it was allowed to stand at room temperature for 10 min, followed by centrifugation at 12,000 rpm for 10 min at 4 °C. The supernatant was discarded and 1 mL of 75% alcohol was added followed by centrifugation at 12,000 rpm for 5 min at 4 °C (elution twice). The supernatant was discarded, and the sample was allowed to stand at room temperature for 10 min. It was then dissolved in 30 μL of DEPC water, and measured spectrophotometrically at 260 nm. RNA purity was determined by measuring the ratio of absorbance readings of the RNA samples at 260 and 280 nm (A260 / A280). The A260/A280 ratio of all RNA samples was between 1.8 and 2.0, indicating that the RNA samples were pure. Each sample was diluted according to the measured concentration and finally diluted to 1000 ng/μL. Each remaining RNA sample was stored at − 80 °C for cDNA synthesis.

The manufacturer’s instructions were followed (Takara Bio, Japan) and all operations were performed on ice. The above diluted 1000 ng/μL RNA sample was mixed with 2 μL of each sample and 2 μL of 5× DNA Eraser Buffer, 1 μL of gDNA Eraser, and RNase Free dH_2_O, and allowed to stand at room temperature for 15 min. Then, 1 μL of Primescript RT Enzyme Mix I, 1 μL of RT Primer Mix, 4 μL of 5 × PrimeScript Buffer 2, 4 μL of RNase Free dH_2_O were added, and placed in the PCR machine at 37 °C for 15 min, 85 °C for 5 seconds.

### Quantitative real-time RT-PCR

The manufacturer’s instructions were followed (Takara Bio, Japan) and all operations were performed on ice. Two microliter of each of the above reverse-transcribed samples were added to 10 μL of TB Green premix Ex Taq II 2×, 0.8 μl upstream primer, 0.8 μL downstream primer, 0.4 μL ROX Reference Dye 50 × and 6 μL The DEPC water such that the total reaction system was 20 μL. The real-time PCR reaction procedure was pre-denaturation at 95 °C for 30 s, and the PCR reaction was carried out for a total of 42 cycles, each cycle being: 95 °C for 5 seconds and 59 °C for 30 s.

Oligonucleotide primers for quantitative RT-PCR analysis of inflammatory factors and Actin housekeeping genes in chicken ileum mucosa are listed in Table 2. Briefly, the CFX Connect Real-Time System (BIO-RAD, USA) and the PrimeScript™RT reagent KIT with gDNA Eraser (Stratagene) were used to amplify and detect equal amounts of total RNA from each sample, and a standard curve was generated using log10 diluted standard RNA. Each analysis was performed in triplicate. To normalize RNA levels between samples within the experiment, the average threshold cycle (Ct) value of the amplified product was calculated by pooling values from all samples in the experiment.

**Table 2:** Primer sequences of RT-PCR

Target	Primer sequence(5′-3′)	Accession no.	Product size (bp)
*claudin-1*	F:TCCAAGCTCACCAAAGAGGG	NM_001013611.2	128
	R:ACCGGTGACAGACTGGTTTC		
*TLR2*	F:TACAGATGCTACTGTGCCTGA	NM_001161650.1	102
	R:CACTTTCCAGTGCCCAAGAG		
*TLR4*	F:TTCCATGGCTTAACGTCGCT	NM_001030693.1	82
	R:AGTGTCCGATGGGTAGGTCA		
*Occludin*	F:TGTGTAAGGCCCACACCTCT	NM_205128.1	92
	R:TGCTCAGGGTACCATTCTGG		
*TNF-α*	F:GAGCGTTGACTTGGCTGTC	XM_015294124.2	64
	R:GAGCGTTGACTTGGCTGTC		
*IL-1β*	F:ACTGGGCATCAAGGGCTA	XM_015297469.1	131
	R:GGTAGAAGATGAAGCGGGTC		
*MUC2*	F:TTCATGATGCCTGCTCTTGTC	XM_421035	93
	R:CCTGAGCCTTGGTACATTCTTGT		
*β-actin*	F:TTGTCCACCGCAAATGCTTC	NM_205518.1	106
	R:AGCCATGCCAATCTCGTCTT		

F was the upstream primer and R was the downstream primer

**Table 3:** Effects of *LP* and Environment on Immune Organ Index chickens after *CP* administration

	1st day			3rd day			10th day		
	Spleen index	Thymus index	Bursa index	Spleen index	Thymus index	Bursa index	Spleen index	Thymus index	Bursa index
SPFC^a^	0.20	0.65	0.42	0.18	0.67	0.38	0.22	0.60	0.41
SPFL	0.16	0.57	0.49	0.19	0.64	0.40	0.18	0.67	0.39
FRC	0.19	0.46	0.53	0.24	0.50	0.45	0.19	0.47	0.47
FRL	0.18	0.51	0.61	0.22	0.61	046	0.19	0.57	0.46
SEM^b^	0.009	0.022	0.025	0.016	0.034	0.021	0.013	0.024	0.034
Main effects^c^									
Environment									
SPF	0.18	0.61	0.45	0.19	0.65	0.39	0.20	0.64	0.40
FRS	0.18	0.49	0.57	0.23	0.55	0.45	0.19	0.52	0.46
*Lactobacillus*									
NLP	0.19	0.55	0.47	0.21	0.59	0.41	0.21	0.54	0.44
LP	0.17	0.55	0.55	0.20	0.62	0.43	0.19	0.63	0.42
*P*-value									
Environment	0.884	0.015	0.030	0.179	0.167	0.163	0.831	0.021	0.385
*Lactobacillus*	0.223	0.875	0.128	0.786	0.600	0.721	0.491	0.066	0.834
environment × *Lactobacillus*	0.500	0.152	0.898	0.586	0.330	0.906	0.469	0.944	0.948

^a^Data of SFPC、SPFL、FRC and FRL were all average values.

^b^SEM meat standard error of mean.

^c^The data for the main effect represented average values.

The following table was the same.

**Table 4:** Effects of environment and *LP* on the duodenal morphology of chickens after *CP* administration

	Villus height (μm)			Crypt depth (μm)			V/C		
	1st day	3rd day	10th day	1st day	3rd day	10th day	1st day	3rd day	10th day
SPFC	954.42	961.49	1001.87	121.52	100.05	107..79	8.56	10.25	9.75
SPFL	1111.29	1017.62	1011.33	85.53	100.26	99.47	13.71	10.62	10.80
FRC	822.79	1007.14	971.91	112.48	145.04	109.76	7.72	7.48	9.25
FRL	1012.06	1067.21	1061.76	115.21	128.37	85.19	9.15	8.92	13.25
SEM	13.059	11.228	9.662	1.950	2.097	1.514	0.244	0.179	0.170
Main effects									
Environment									
SPF	1036.66	983.39	1006.57	102.65	100.13	103.65	11.26	10.39	10.27
FRS	926.03	1040.66	1010.42	113.97	135.74	99.23	8.50	8.28	10.96
*Lactobacillus*									
NLP	895.26	981.96	986.99	117.46	120.23	108.77	8.18	9.01	9.50
LP	1064.59	1048.17	1032.94	99.49	117.57	93.35	11.57	9.57	11.85
*P*-value									
Environment	0.000	0.035	0.597	0.009	0.000	0.043	0.000	0.000	0.005
*Lactobacillus*	0.000	0.010	0.011	0.000	0.051	0.000	0.000	0.012	0.000
Environment × *Lactobacillus*	0.536	0.930	0.038	0.000	0.045	0.008	0.000	0.135	0.000

V/C represented the ratio of the height of the villi to the depth of the crypt, the same as the table below

**Table 5:** Effects of environment and *LP* on the jejunal morphology of chickens after CP administration

	Villus height (μm)			Crypt depth (μm)			V/C		
	1st day	3rd day	10th day	1st day	3rd day	10th day	1st day	3rd day	10th day
SPFC	824.31	988.71	953.85	91.69	93.65	94.68	9.18	11.46	10.78
SPFL	973.34	1057.00	957.08	70.47	79.29	84.60	14.45	14.48	11.74
FRC	932.66	975.54	907.46	139.05	114.79	102.65	6.99	9.17	9.26
FRL	872.28	836.24	843.10	98.61	95.90	83.80	9.08	9.13	10.50
SEM	10.719	14.326	7.164	1.617	1.805	1.376	0.167	0.249	0.151
Main effects									
Environment									
SPF	890.55	1014.05	955.43	82.26	88.325	89.74	11.52	12.58	11.25
FRS	903.96	906.37	875.28	119.83	105.41	93.22	7.98	9.15	9.88
Lactobacillus									
NLP	885.14	982.34	930.98	118.28	103.87	98.61	7.95	10.35	10.03
LP	913.53	922.30	899.69	87.13	89.43	84.19	11.27	11.22	11.12
*P*-value									
Environment	0.865	0.000	0.000	0.000	0.000	0.194	0.000	0.000	0.000
Lactobacillus	0.040	0.216	0.034	0.000	0.000	0.000	0.000	0.003	0.000
Environment × Lactobacillus	0.000	0.000	0.019	0.003	0.531	0.112	0.000	0.002	0.646

**Table 6:** Effects of environment and *LP* on the ileal morphology of chickens after *CP* administration

	Villus height (μm)			Crypt depth (μm)			V/C		
	1st day	3rd day	10th day	1st day	3rd day	10th day	1st day	3rd day	10th day
SPFC	531.29	478.77	547.09	64.04	60.28	75.80	8.39	8.15	7.79
SPFL	702.84	504.61	570.35	65.72	59.20	68.50	11.02	8.61	8.79
FRC	750.26	707.70	648.94	92.14	106.34	88.02	8.49	7.22	7.79
FRL	759.81	518.52	662.89	88.42	90.50	69.06	9.09	6.02	9.88
SEM	8.757	9.267	10.035	1.080	1.556	1.305	0.138	0.133	0.179
Main effects									
Environment									
SPF	610.85	494.32	558.56	64.82	59.63	72.20	9.61	8.42	8.29
FRS	755.00	602.30	655.62	90.29	97.51	78.95	8.79	6.55	8.79
*Lactobacillus*									
NLP	640.03	608.99	593.04	77.99	86.48	81.31	8.44	7.62	7.79
LP	733.00	511.89	610.75	77.74	75.59	68.75	10.00	7.25	9.17
P-value									
Environment	0.000	0.000	0.000	0.000	0.000	0.015	0.001	0.000	0.129
*Lactobacillus*	0.000	0.000	0.357	0.637	0.007	0.000	0.000	0.164	0.000
Environment × *Lactobacillus*	0.000	0.000	0.818	0.213	0.018	0.026	0.000	0.002	0.129

**Table 7:** Effects of *LP* and environment on biochemical parameters in serum of chickens after *CP* administration

	TP (g/L)			ALB (g/L)			T-SOD (U/mL)			AKP (Gold unit / 100 ml)			GOT (IU/L)		
	1st	3rd	10th	1st	3rd	10th	1st	3rd	10th	1st	3rd	10th	1st	3rd	10th
SPFC	36.24	36.51	35.15	23.24	22.31	20.76	215.35	201.58	183.21	400.28	429.01	278.80	112.81	39.31	36.15
SPFL	28.35	29.82	30.26	19.99	14.65	19.70	157.82	148.21	172.31	293.35	270.47	345.13	94.71	18.45	13.09
FRC	40.70	34.00	48.21	22.79	21.31	22.54	168.76	170.59	194.69	647.49	788.63	656.74	73.38	36.59	29.51
FRL	30.97	34.71	33.39	19.91	18.99	20.84	157.82	151.64	165.56	305.02	524.97	362.01	32.04	24.97	17.05
SEM	0.899	0.425	1.630	0.306	0.825	0.403	6.250	4.795	3.166	36.526	53.607	38.121	4.869	1.265	1.798
Main effects															
Environment															
SPF	32.30	33.16	32.71	21.61	18.48	20.23	186.58	174.89	177.76	346.81	349.74	311.96	103.76	28.88	24.62
FRS	35.83	34.35	40.80	21.35	20.42	21.69	163.00	161.11	180.13	476.26	656.80	509.38	52.71	30.78	23.28
*Lactobacillus*															
NLP	38.47	35.26	41.68	23.02	22.08	21.65	192.05	186.08	188.95	523.88	608.82	467.77	93.10	37.95	32.36
LP	29.66	32.26	31.82	19.95	16.82	20.27	157.53	149.92	168.94	299.19	397.71	353.57	63.38	21.71	15.35
*P*-value															
Environment	0.085	0.199	0.038	0.679	0.275	0.108	0.096	0.189	0.718	0.114	0.021	0.032	0.001	0.475	0.717
*Lactobacillus*	0.001	0.008	0.016	0.001	0.013	0.125	0.025	0.005	0.013	0.015	0.084	0.173	0.016	0.000	0.001
Environment × *Lactobacillus*	0.623	0.002	0.166	0.770	0.184	0.702	0.103	0.110	0.188	0.146	0.637	0.045	0.267	0.106	0.171

**Table 8:** Effects of *LP* and environment on immune parameters in serum of chickens after *CP* administration

	IgA (mg/mL)			IgM (mg/mL)			IgG (mg/mL)		
	1st	3rd	10th	1st	3rd	10th	1st	3rd	10th
SPFC	5.87	3.00	3.12	5.10	4.01	3.07	66.00	52.08	35.96
SPFL	12.20	4.81	8.55	6.04	4.48	4.99	90.59	58.32	79.84
FRC	1.97	2.44	3.06	4.04	4.81	4.29	57.48	36.88	37.18
FRL	5.24	5.70	5.28	4.77	4.93	4.77	76.47	84.47	61.40
SEM	1.689	0.474	0.350	0.239	0.175	0.179	5.500	5.924	5.096
Main effects									
Environment									
SPF	9.04	3.90	5.84	5.58	4.25	4.03	76.54	55.20	54.77
FRS	3.60	4.07	4.17	4.40	4.87	4.53	66.97	60.68	49.29
Lactobacillus									
NLP	3.92	2.72	3.09	4.57	4.41	3.68	61.74	43.63	36.64
LP	8.72	5.25	6.91	5.40	4.70	4.88	82.52	72.85	68.32
*P*-value									
Environment	0.146	0.867	0.044	0.041	0.114	0.196	0.326	0.651	0.413
Lactobacillus	0.193	0.028	0.001	0.120	0.424	0.010	0.073	0.039	0.005
Environment × Lactobacillus	0.663	0.468	0.051	0.839	0.637	0.078	0.804	0.103	0.353

**Table 9:** Effects of *LP* and environment on tight junction protein in ileum mucosa of chickens after *CP* administration

	MUC2			Claudin			Occludin		
	1st	3rd	10th	1st	3rd	10th	1st	3rd	10th
SPFC	1.00	1.00	1.00	0.66	1.00	1.45	0.74	1.00	0.92
SPFL	1.16	1.66	1.40	1.00	1.53	2.12	1.00	3.52	1.29
FRC	0.64	0.63	0.49	1.07	0.57	0.72	0.41	0.40	0.28
FRL	1.45	1.90	1.22	2.19	1.56	1.00	0.94	1.90	1.00
SEM	0.076	0.137	0.142	0.324	0.163	0.422	0.270	0.531	0.310
Main effects									
Environment									
SPF	1.07	1.25	1.15	0.85	1.20	1.78	0.88	1.94	1.01
FRS	1.04	1.17	0.91	1.63	1.06	0.90	0.64	1.15	0.73
*Lactobacillus*									
NLP	0.84	0.84	0.81	0.89	0.84	1.09	0.58	0.73	0.60
LP	1.30	1.78	1.30	1.59	1.55	1.42	0.98	2.60	1.11
*P*-value									
Environment	0.820	0.820	0.254	0.238	0.547	0.299	0.729	0.317	0.475
*Lactobacillus*	0.011	0.005	0.072	0.278	0.041	0.587	0.479	0.082	0.400
Environment × Lactobacillus	0.060	0.287	0.569	0.559	0.496	0.822	0.808	0.644	0.788

**Table 10:** Effects of *LP* and environment on inflammatory factors of ileum mucosa of chickens after *CP* administration

	IL-1β			TNF-α			TLR2			TLR4		
	1st	3rd	10th	1st	3rd	10th	1st	3rd	10th	1st	3rd	10th
SPFC	1.00	1.00	1.00	1.00	1.00	2.21	1.00	1.00	1.00	1.00	1.00	1.00
SPFL	0.60	0.69	0.55	0.80	0.69	0.73	0.52	0.45	0.62	0.68	0.46	0.54
FRC	0.67	1.72	1.25	1.71	1.15	1.00	0.83	0.59	1.90	0.83	1.00	1.51
FRL	0.53	1.02	0.73	0.81	0.48	0.79	0.61	0.38	0.60	0.71	0.31	0.58
SEM	0.147	0.140	0.108	0.122	0.093	0.132	0.141	0.197	0.258	0.130	0.204	0.147
Main effects												
Environment												
SPF	0.80	0.88	0.83	0.93	0.86	1.47	0.79	0.75	0.86	0.86	0.80	0.83
FRS	0.61	1.32	0.99	1.19	0.86	0.92	0.72	0.51	1.25	0.78	0.65	1.04
Lactobacillus												
NLP	0.82	1.27	1.09	1.27	1.07	1.45	0.93	0.79	1.34	0.92	1.00	1.19
LP	0.56	0.88	0.64	0.81	0.60	0.76	0.56	0.42	0.61	0.69	0.38	0.56
*P*-value												
Environment	0.510	0.086	0.349	0.171	0.860	0.055	0.880	0.552	0.416	0.795	0.859	0.372
Lactobacillus	0.372	0.099	0.051	0.046	0.022	0.010	0.235	0.352	0.135	0.404	0.163	0.039
Environment × Lactobacillus	0.667	0.505	0.873	0.176	0.347	0.037	0.664	0.667	0.398	0.709	0.865	0.438

### Data analysis

All data were analyzed with SPSS version17.0 (SPSS Inc., Chicago, IL). A significance level of 0.05 was used. The data of intestinal lesion score were analyzed by one-way ANOVA, and were subjected to grouped table of the GraphPad Prism 5 (GraphPad Software, Inc., CA, USA). Other data were analyzed by two-factorial analysis of variance to examine the main effects of probiotic and challenge, and their interaction using general linear model procedure SPSS 17.0. When a significant interaction between the main effects was observed, Duncan’s multiple comparison was used to compare the differences among the four groups. Results in the tables were given as the mean and pooled SEM.

## Data Availability

The dataset supporting the conclusions of this article is included within the article and additional Tables 3, 4, 5, 6, 7, 8, 9 and 10 and Fig. 1.

## Abbreviations

AKP: Alkaline phosphatase
ALB: Albumin
*CP*: *Clostridium perfringens*
FRS: Free-range system
GOT: Glutamic oxaloacetic transaminase
IgA: Immunoglobulin A
IgG: Immunoglobulin G
IgM: Immunoglobulin M
*LP*: *Lactobacillus plantarum*
NE: Necrotic enteritis
NLP: No *Lactobacillus plantarum*
SPF: Specific pathogen free
SPFC: Specific pathogen free environment + base diet
SPFL: Specific pathogen free environment + *Lactobacillus plantarum*
TP: Total protein
T-SOD: Total superoxide dismutase
V/C: The ratio of the height of the villi to the depth of the crypt
